# BRIDGING THE DIGITAL DIVIDE: FACTORS INFLUENCING EHEALTH USE AMONG HOMEBOUND OLDER ADULTS DURING THE PANDEMIC

**DOI:** 10.1093/geroni/igae098.2325

**Published:** 2024-12-31

**Authors:** Joonhyeog Park, BoRin Kim, Joonyoung Cho, Sojung Park

**Affiliations:** School of Social Policy and Practice, University of Pennsylvania, Philadelphia, Pennsylvania, United States; University of New Hampshire, Durham, New Hampshire, United States; University of Hawaii, Hawaii, United States; Washington University in St. Louis, St. Louis, Missouri, United States

## Abstract

The pandemic has increased the use of eHealth. However, homebound older adults may face a significant digital divide and need support in adopting eHealth. This study investigates to what extent and how access to devices for information and communication technologies (ICTs), previous online experience, and ICT training are associated with eHealth use during the pandemic. Data came from the COVID-19 survey of the National Health and Aging Trend Study. The sample was restricted to homebound older adults aged 70+ who rarely or never left home, or left home with difficulty or assistance (N=591). Hierarchical multinomial logistic regression was used to examine the influence of access to ICT devices, online experience, and ICT training with or without assistance on eHealth engagements including non-users, patient portal users, video telehealth users, and users of both services. Findings indicate that access to ICT devices was positively associated with all forms of eHealth use: patient portal (RRR=3.69, p<.05), video telehealth (RRR=2.56, p<.05), and both (RRR=3.81, p<.05). However, after adding previous online experience and ICT training into the analyses, access to ICT devices was not significant. While previous online experience was a significant predictor for the use of patient portal (RRR=11.97, p<.001), ICT training with assistance significantly increased the probability of the use of video telehealth and both forms of eHealth. This study highlights that access to ICT devices alone does not address eHealth disparities among homebound older adults. Educational interventions and strategies that encourage online engagement are crucial to bridge this gap.

